# Nanometer-resolution in situ structure of the SARS-CoV-2 postfusion spike protein

**DOI:** 10.1073/pnas.2112703118

**Published:** 2021-11-15

**Authors:** Linhua Tai, Guoliang Zhu, Minnan Yang, Lei Cao, Xiaorui Xing, Guoliang Yin, Chun Chan, Chengfeng Qin, Zihe Rao, Xiangxi Wang, Fei Sun, Yun Zhu

**Affiliations:** ^a^National Key Laboratory of Biomacromolecules, CAS Center for Excellence in Biomacromolecules, Institute of Biophysics, Chinese Academy of Sciences, Beijing 100101, China;; ^b^School of Life Sciences, University of Chinese Academy of Sciences, Beijing 100049, China;; ^c^Division of Medicinal Chemistry and Pharmacognosy, College of Pharmacy, The Ohio State University, Columbus, OH 43210;; ^d^State Key Laboratory of Pathogen and Biosecurity, Beijing Institute of Microbiology and Epidemiology, Beijing 100071, China;; ^e^Department for Disease Control and Prevention, Guangzhou Laboratory, Guangzhou 510320, China;; ^f^Department of Biomedical Device, Bioland Laboratory, Guangzhou 510005, China;; ^g^Center for Biological Imaging, Institute of Biophysics, Chinese Academy of Sciences, Beijing 100101, China

**Keywords:** cryo-electron tomography, postfusion state, SARS-CoV-2, spike protein, subtomogram analysis

## Abstract

The spike protein of severe acute respiratory syndrome coronavirus 2 (SARS-CoV-2) mediates membrane fusion to allow entry of the viral genome into host cells. To understand its detailed entry mechanism and develop a specific entry inhibitor, in situ structural information on the SARS-CoV-2 spike protein in different states is urgent. Here, by using cryo-electron tomography, we observed both prefusion and postfusion spikes in β-propiolactone–inactivated SARS-CoV-2 virions and solved the in situ structure of the postfusion spike at nanometer resolution. Compared to previous reports, the six-helix bundle fusion core, the glycosylation sites, and the location of the transmembrane domain were clearly resolved. We observed oligomerization patterns of the spikes on the viral membrane, likely suggesting a mechanism of fusion pore formation.

## Significance

Severe acute respiratory syndrome coronavirus 2 (SARS-CoV-2) is a severe threat to public health and the global economy. Its spike protein is responsible for the membrane fusion and is thus a major target for vaccine and drug development. Our study presents the in situ structure of the spike protein in the postfusion state with higher resolution, giving further insights into the design of a viral entry inhibitor. Our observation of the oligomerization states of spikes on the viral membrane implies a possible mechanism of membrane fusion for viral infection.

Over the past two decades, several zoonotic coronavirus (CoV) diseases have emerged and posed a devastating threat to global public health and the economy, such as severe acute respiratory syndrome (SARS) (1), Middle East respiratory syndrome (MERS) (2), and COVID-19 (3). As of this writing, COVID-19 has more than 229 million confirmed cases and has caused 4.7 million deaths worldwide, with rapidly increasing numbers. This pneumonia epidemic was caused by a novel coronavirus named SARS coronavirus 2 (SARS-CoV-2), a β-coronavirus, with a genomic sequence that is closely related to SARS-CoV. SARS-CoV-2 is an enveloped, positive-sense single-stranded RNA virus with an ∼30-kb genome (4). Given the current pandemic situation, understanding the structure of SARS-CoV-2 as well as its infection process is very important for vaccine development and drug discovery.

The SARS-CoV-2 genome encodes three viral surface proteins: the spike (S) glycoprotein, envelope (E) protein, and membrane (M) protein. During the infection process, the trimeric S glycoprotein is cleaved by host proteases (4, 5) to produce two functional subunits: The N-terminal S1 subunit is responsible for receptor recognition, and the C-terminal S2 subunit is responsible for membrane fusion (6). Mediated by receptor binding and proteolytic activation, the S1 subunit falls off, and the S2 subunit undergoes extensive and irreversible conformational changes to insert its hydrophobic fusion peptide (FP) into the target cell membrane. Subsequently, two heptad repeat regions of the S2 subunit, heptad repeat 1 (HR1) and heptad repeat 2 (HR2), form a stable six-helix bundle (6-HB) fusion core to bring together the viral and cellular membranes, leading to colocalization of the FP and the transmembrane (TM) region at the same end to form the fusion pore (7). Thus, the S protein is one of the major targets for developing vaccines and antiviral drugs.

After the outbreak of COVID-19, the in vitro structures of SARS-CoV-2 S in the prefusion state were promptly solved using single-particle cryo-electron microscopy (cryo-EM) (8, 9) and X-ray crystallography (7, 10, 11). Soon afterward, the in situ structures of S in the prefusion state were revealed by cryo-electron tomography (cryo-ET) and cryo-subtomogram averaging (cryo-STA) (12–14), uncovering the distribution of different conformational states as well as the native glycosylation sites. However, how the S protein is activated to induce membrane fusion with its host is less understood. The structure of S in the postfusion state would provide an important clue to investigate the fusion mechanism. The high-resolution structure of recombinant S in the postfusion state has been reported by Cai et al. (15), but this in vitro study failed to determine how the postfusion S proteins organize on the membrane. Previous in situ studies (12, 13, 16) explored this question but yielded limited information, due to the poor quality of the density map. In addition, we previously showed that the recombinant 6-HB fusion core of S in the postfusion state would be an effective target for the design of viral fusion inhibitors (7), which needs to be further validated by a higher-resolution structure and glycosylation information of in situ S in the postfusion state.

In the present work, we utilized cryo-ET and cryo-STA to study the structure of SARS-CoV-2 viruses that were inactivated by β-propiolactone (BPL). We solved the in situ structures of S in both the prefusion and postfusion states with resolutions of 12.9 and 10.9 Å, respectively. In addition to visualizing the TM region and glycosylation sites, we found that our previous crystal structure of the recombinant 6-HB fusion core fits well to the density map. In addition, we observed oligomerization of postfusion Ss on the viral membrane, suggesting a mechanism of S-induced membrane fusion. Our study will facilitate a better understanding of the SARS-CoV-2 fusion mechanism and be beneficial for viral entry inhibitor development.

## Results

### Cryo-ET Analysis of the Inactivated SARS-CoV-2 Virus.

**Fig. 1.**
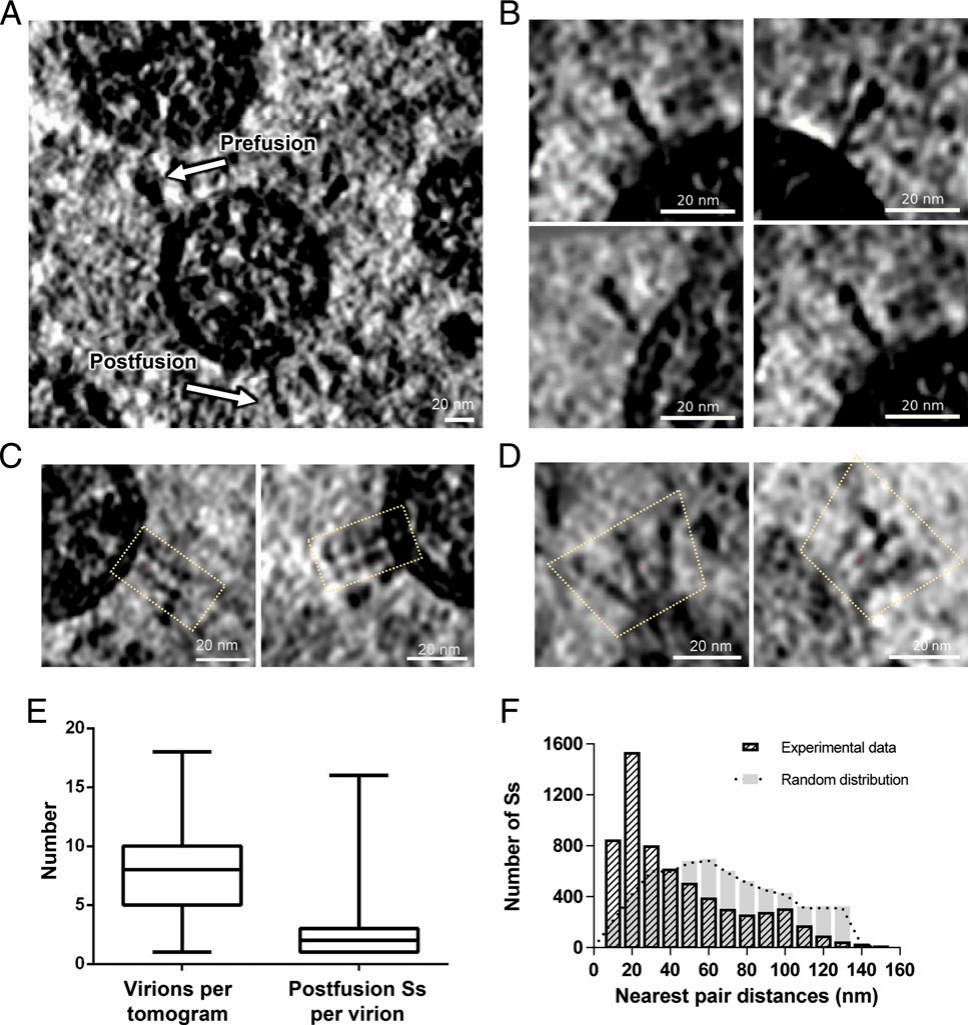
Cryo-ET of inactivated SARS-CoV-2 virions. (*A*) Slice view of tomographic reconstructions of BPL-inactivated SARS-CoV-2 virions. White arrows indicate Ss in the prefusion and postfusion states. (Scale bar, 20 nm.) (*B*) Selected slices of separate postfusion Ss. (*C* and *D*) Selected slices of oligomerized postfusion Ss with side-by-side (*C*) and branching (*D*) patterns. Dotted outlines indicate the adjacent postfusion Ss. All tomograms were deconvolved using Warp (17) and are displayed using IMOD (38). (*E*) Statistics of virion numbers per tomogram and numbers of postfusion Ss per virion. (*F*) Histograms of nearest pair distances for postfusion Ss in the experimental data and in the simulated data with random distributions.

We propagated SARS-CoV-2 virions into Vero cells and purified the viral particles in a biosafety level 3 (BSL-3) laboratory. The purified virus was inactivated with BPL and imaged by cryo-ET in a BSL-2 laboratory. In the reconstructed tomograms, we observed a typical coronavirus morphology of SARS-CoV-2 virions with diameters ranging from 80 nm to 120 nm (Fig. 1*A*). Inside each virion, the ribonucleoprotein complexes were tightly packed, with a diameter of ∼15 nm. From the deconvoluted tomograms using Warp (a computer software for cryo-EM data processing) (17), we could clearly visualize most Ss that were ready for subsequent particle picking. Both the prefusion and postfusion states of S were observed (Fig. 1 *A* and *B*), as reported previously (16), which was in line with the fact that cleavage of S had occurred during the sample preparation (*SI Appendix*, Fig. S1).

Previous studies have argued that the percentages of S in the prefusion and postfusion states are related to viral inactivation methods. The majority of prefusion S was from formaldehyde-fixed samples (12), while a great portion of postfusion S appeared to be from the BPL-inactivated sample, with a ratio of up to 66 to 81.3% (16, 18). In our sample, we further investigated the ratio of prefusion to postfusion Ss. We picked all possible S particles by combining the template matching approach with the manual method. We averaged the maps of both prefusion and postfusion Ss to generate a reference for subsequent three-dimensional (3D) classification, which showed that 42% of the particles were classified into the prefusion state, and 48% were classified into the postfusion state (*SI Appendix*, Fig. S2). Thus, the populations of prefusion and postfusion Ss were similar in our BPL-inactivated sample, which was different from a previous report (16).

A recent study showed that prefusion S exhibits a flexible orientation with respect to the viral membrane, tilting from the vertical axis to the viral membrane at a range of 50° (12). This structural feature could help prefusion S seek and bind to the ACE2 receptor of the target cell. In contrast, by visual inspection, we found that a great portion of the postfusion Ss appeared perpendicular to the viral membrane, which suggested that the conformation of the postfusion S has a stable membrane proximal external region or a stable TM region. In addition to the dispersed postfusion Ss on the viral membrane (Fig. 1*B*), we also observed that some postfusion Ss oligomerized in parallel (Fig. 1*C*) or in branches (Fig. 1*D*). We statistically determined the postfusion S on the viral membrane and found that the distribution of the postfusion S was sparse, with three Ss per virion on average (Fig. 1*E*). However, by inspecting all pairs of postfusion Ss in the same virions and calculating the pair distances, we found that many pairs had distances of ∼20 nm or less (Fig. 1*F*), implying potential clustering behavior of postfusion Ss. In order to validate this observation, we generated a simulated dataset, in which the numbers of viruses and Ss on each virus were all kept the same as the experimental data, but the postfusion Ss were randomly distributed on sphere-shaped virus. Using the same calculation method as for the nearest pair distance, the randomly placed Ss had no clustering effect, showing a normal distribution pattern with a center of 60 nm (Fig. 1*F*). This suggested that the clustering peak of postfusion Ss in our experimental dataset was statistically significant.

### Subtomogram Analysis of SARS-CoV-2 Postfusion S.

We then performed subtomogram analysis from a total of 15,525 selected S particles (*SI Appendix*, Figs. S2 and S3). After 3D classification and autorefinement, we obtained an in situ structural map of prefusion S with C3 symmetry at a resolution of 12.9 Å according to the gold standard Fourier shell correlation (FSC) coefficient at 0.143 (*SI Appendix*, Fig. S2). Our in situ structure of prefusion S was similar to those obtained in previous reports (12, 13). In the present study, we focused on postfusion S showing the nail shape on the viral membrane.

**Fig. 2.**
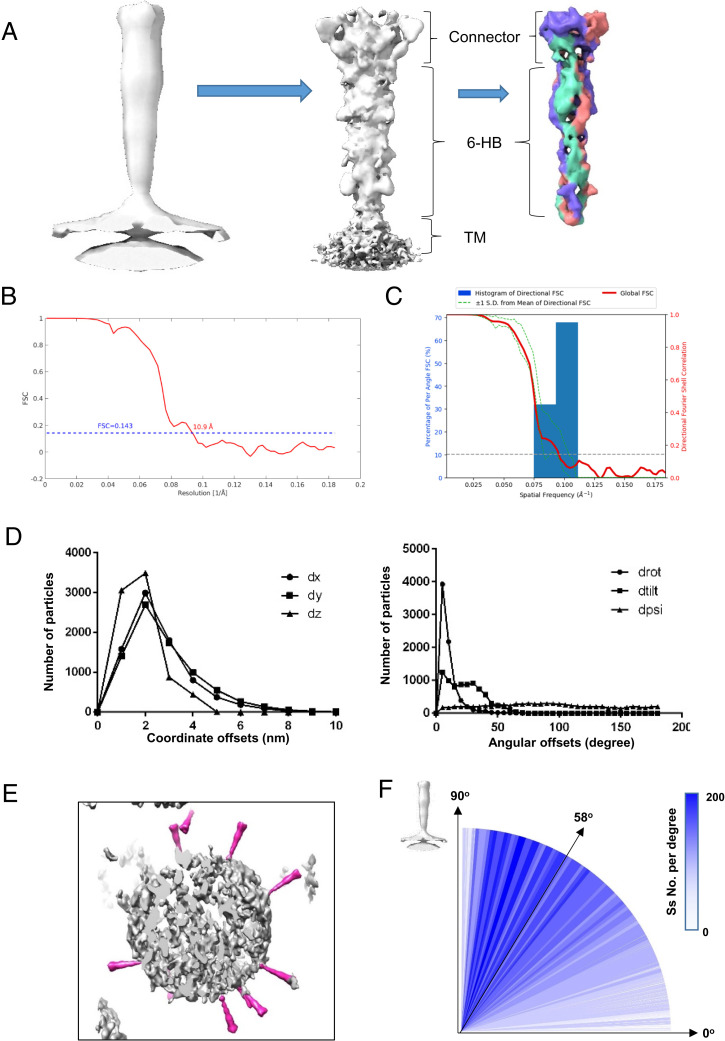
Subtomogram averaging of the SARS-CoV-2 postfusion S. (*A*) Subtomogram averaging procedure, including the initial average of all particles at a binning level of eight (*Left*), the refined map at a binning level of two (*Middle*), and the postprocessed map with a local mask at the extracellular region at a binning level of two (*Right*). (*B*) The gold standard FSC curves for the final averaged map of postfusion S. (*C*) The directional FSC of the final averaged map of postfusion S. (*D*) Statistics of the coordinate and Euler angle changes before and after alignment during image processing. The coordinate offsets on the *x, y, z* axes are defined as dx, dy, and dz, while the angular offsets at three Euler angles are defined as drot, dtilt, and dpsi. (*E*) Plot back of the averaged map onto the original tomogram, showing relative orientations of postfusion Ss to the virus membrane. (*F*) Distribution of the tilting angles of postfusion Ss relative to viral envelope.

We utilized different approaches to align the particles of postfusion Ss by trying local or global searches of orientations with C1 or C3 symmetries. We found that only local searches with C3 symmetry with restriction of the Euler angles that had prior values during particle picking (*SI Appendix*, Fig. S2) could yield a high-resolution (10.9 Å) map according to the gold standard FSC coefficient at 0.143 (Fig. 2 *A* and *B*). From the averaged map of postfusion S, we clearly distinguished the head region (connector), stalk region (6-HB), and TM region. The three S protomers could also be distinguished from the map with a higher threshold (Fig. 2*A*). The directional FSC analysis confirmed that there was no preferred orientation in our dataset (Fig. 2*C*).

To further validate the results of our alignment and refinement, we inspected the shift and orientation changes of particles before and after alignment. We found that most particles shifted less than 4 nm compared to their manually picked coordinates (Fig. 2*D*). For the three Euler angles, the first one (rot. angle) exhibited the smallest change with a mean value of 7.38°, changes in the tilt angle had a mean value of 21.2°, and the psi angle showed no correlation with the starting value (Fig. 2*D*). Manual picking could identify only the first two Euler angles (rot. and tilt), and our observation of shift and orientation changes agreed well with this fact.

After plotting the refined map back onto the raw tomogram with refined coordinates and orientations, we found that all the postfusion Ss were in the rational place on the viral envelope (Fig. 2*E*). Moreover, the relative angles of postfusion Ss to virion were calculated, indicating that most Ss had tilting angles larger than ∼45° relative to the envelope, and the median value is ∼58° (Fig. 2*F*). These statistics agreed well with visual inspections and the plot back result. Comparing with previous reports for tilting angles of prefusion Ss (12, 13), it seems that Ss in postfusion state tend to be more perpendicular to the viral envelope. In addition, to validate whether the orientations of postfusion Ss were affected by air–water interface (AWI), we calculated the distributions of postfusion Ss in the *z* axis of original tomograms, and found that more than 90% postfusion Ss were far away from AWI (*SI Appendix*, Fig. S3*C*). This result further proved the reliability of our findings.

### In Situ Structure of SARS-CoV-2 Postfusion S.

**Fig. 3.**
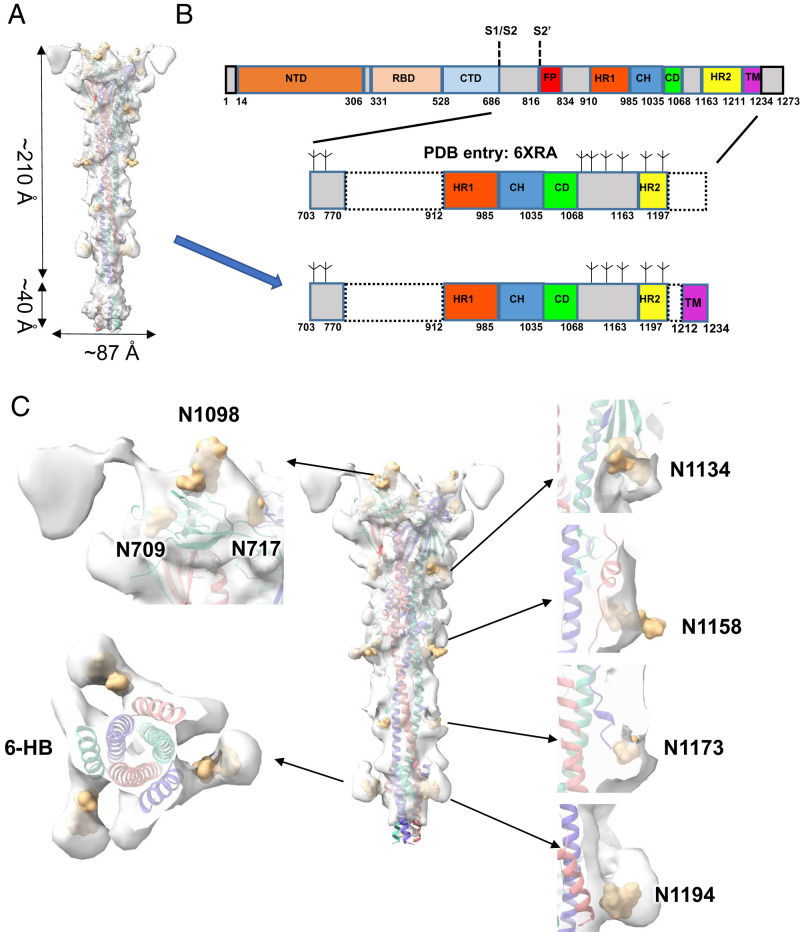
In situ structure of the SARS-CoV-2 postfusion S. (*A*) Geometry of postfusion S on the membrane. This map was generated by merging the TM region of the full averaged map onto the higher-resolution map from focused alignment on the extracellular domains. (*B*) Domain arrangement of full-length S protein and the modeled parts of the published structure (PDB entry: 6XRA) and our model. The potential cleavage sites and glycosylation sites are indicated. (*C*) Superimposition of our model with the merged map mentioned in *A*, showing the glycosylation sites and distinguished 6-HB domains. The observed glycosylation sites are shown in burlywood. Three S protomers are shown in medium slate blue, light coral, and medium turquoise.

The in situ structure of postfusion S of SARS-CoV-2 has a length of ∼210 Å and a width of ∼87 Å at the ectodomain (Fig. 3*A* and Movie S1). Its overall architecture resembles the previously reported recombinant postfusion S (Protein Data Bank [PDB] entry: 6XRA) (15) with an unusually long (>180 Å) and rigid 6-HB formed by the HR1 and HR2 domains (Fig. 3*A* and *SI Appendix*, Fig. S4). On this basis, we built an in situ structural model with an extension at the TM domain (Fig. 3 *A* and *B*). Compared to previous recombinant structures with indistinct TM locations (15), the TM domain can be easily discerned in our in situ map beneath the HR2 trimer (Fig. 3*A* and *SI Appendix*, Fig. S4). Since the local density map of the TM region was not good enough to distinguish individual helices, we fitted the predicted model of the TM domain (Trp1212 to Leu1234) in the map (Fig. 3*B*). In addition, the three helices of HR2 could be clearly distinguished from the helices of HR1 in the 6-HB region (Fig. 3*C* and *SI Appendix*, Fig. S5). Compared to the reported in situ structures of postfusion S of SARS-CoV-2 (Electron Microscopy Data Bank (EMD) accession codes EMD-11627 and EMD-30428) (13, 16), our map exhibits much better quality at the regions of the connector, 6-HB, and TM domains to disclose more structural details (*SI Appendix*, Fig. S4). Based on our structure, an updated model of how the S protein changes its conformation from prefusion to postfusion during viral infection was proposed (Movie S2).

Glycosylation has extensive roles in viral pathogenesis, such as immune evasion, by shielding specific epitopes from neutralizing antibodies. Based on a previous study (8), there are 22 *N*-linked glycosylation sites in each chain of the SARS-CoV-2 S protein, of which 16 sites are situated before the FP domain and 6 are situated after. According to the covered sequence region of the S2 subunit in our model, there were eight *N*-glycosylation sites (N709, N717, N1074, N1098, N1134, N1158, N1173, and N1194) (15). Among them, we observed seven *N*-glycosylation sites (N709, N717, N1098, N1134, N1158, N1173, and N1194) with clear densities in our map (Fig. 3*C*). These glycosylation sites are similar to those previously reported from in situ and in vitro studies of postfusion Ss (13, 15, 16), providing additional evidence that these posttranslational modifications are highly conserved and widespread in various SARS-CoV-2 strains and should play important roles in viral infections and immune responses. We did not observe an obvious density for *N*-glycosylation at N1074, suggesting either no glycosylation or a low level of glycosylation for this site in our sample. Previous studies have reported that glycosylation at N717 and N1074 mainly incorporates oligomannose, while N1098, N1134, N1158, N1173, and N1194 glycosylation mainly incorporates complex-type glycans (15). In our subtomogram-averaged map, we found that the densities of complex-type glycans were more obvious than those of oligomannose (Fig. 3*C*).

### In Situ Oligomerization of SARS-CoV-2 Postfusion S.

**Fig. 4.**
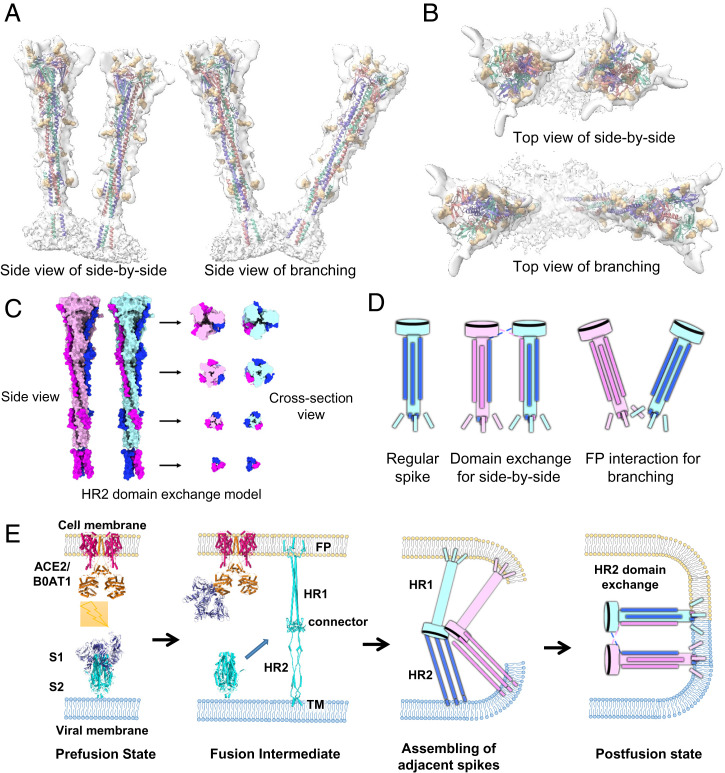
Oligomerization arrangement of the in situ SARS-CoV-2 postfusion S. (*A* and *B*) Side-by-side (*A*) and branching (*B*) arrangements of postfusion Ss. The orientation of each spike was determined according to the final refined parameters. Coloring scheme is the same as that in Fig. 3*C*. (*C*) Possible model of HR2 domain exchange in the side-by-side oligomerization state. The HR2 domains of the two Ss are colored magenta and blue. The other parts of the two Ss are colored pink and cyan. (*D*) Models of in situ postfusion S in the regular state, side-by-side state with HR2 domain exchange, and branching state with FP interaction. (*E*) A scheme of the SARS-CoV-2 S transition from the prefusion to the postfusion state during viral infection and fusion pore formation with the side-by-side oligomerization of Ss involved.

One important step during viral infection of enveloped viruses is the formation of fusion pores between viral and cell membranes, which makes entry of the viral genome into the host cell possible. SARS-CoV-2 is triggered by the conformational change of the S2 subunit from the prefusion to postfusion state through a “jackknife” transition, which effectively brings viral and cellular membranes into close proximity ready for fusion (Movie S2). During particle picking, we noticed that two assembly patterns of postfusion Ss existed on the viral membrane: One type showed the Ss standing parallel to each other (Fig. 1*C*), and the other type, jointed at the root, was heading out in different directions (Fig. 1*D*). We plotted back all refined particles into the reconstructed volumes, fitted the final model into the maps, and then managed to clearly observe the organizing pattern of these specific Ss with the side-by-side and branching patterns (Fig. 4 *A* and *B*). It is worth noting that this oligomerization pattern has never been found for the purified S protein or in situ prefusion state of SARS-CoV-2 S.

For the side-by-side pattern, the postfusion Ss are parallel and close to each other (<10 nm for the 6-HB domain), and their connector domains are closer, almost close enough to form direct contact (Fig. 4 *A* and *B*). Based on this observation, we proposed a possible mechanism by which Ss may interact with each other through flexible HR2 domains in a domain exchange manner (Fig. 4*C*). During the folding process of 6-HB from the prefusion state to the postfusion state, the HR2 domain seeks a neighboring HR1 domain for binding. If there are other Ss around, it might be likely that the HR2 domain could bind to the adjacent HR1 trimer to form a domain exchange conformation (Fig. 4 *C* and *D*). In this way, each postfusion S could maintain an intact trimer after conformational change but will stand close and parallel to its partner. For the branching pattern, Ss stand in branches with jointed roots in the TM region, and this state may be due to the oligomerization of their FPs or even TM domains (Fig. 4*D*). These two types of organization patterns will significantly enhance the local abundance of postfusion S on the viral membrane, which may play an important role in the formation of fusion pores (Fig. 4*E*).

## Discussion

Enveloped viruses use specialized protein machinery to bring viral and cellular membranes into close proximity for membrane fusion. Despite extensive studies on protein machinery and its fusion activity, the molecular mechanism by which viral and cellular membranes promote fusion is poorly understood, especially fusion pore formation. Some models of membrane fusion leading to viral infection have been proposed, and protein machinery oligomerization might facilitate the formation of fusion pores (19–21). On this basis, we proposed a possible model of SARS-CoV-2 membrane fusion and infection (Fig. 4*E*). After the interaction between the S1 subunit and the human ACE2 receptor, the S2 subunit is exposed and undergoes a conformational change to insert FPs into the target cell membrane. At this point, if there are multiple Ss nearby, they could oligomerize to form side-by-side or branching structures. This kind of oligomeric state will lead to an increased concentration of Ss in the local region, possibly enhancing the localized destabilization of lipid bilayers and leading to a more efficient formation of viral fusion pores.

Generally, the intramolecular interactions are more favorable than intermolecular interactions. If our proposed model for oligomerizations of postfusion Ss is correct, the prefusion Ss should be close enough to each other in advance, before the rearrangement of S2 domain, for domain swap to take place. By also calculating the nearest pair distance of prefusion Ss in our dataset, we found a peak distance of around ∼30 nm (*SI Appendix*, Fig. S3*B*). This was slightly longer than the peak distance of postfusion Ss (Fig. 1*F*), but it may be speculated that those prefusion Ss close to each other made it possible for the rearrangement of the S2 domain in the postfusion state. In addition, the similar oligomerization patterns of postfusion Ss have been exhibited but not discussed in a previous report (16). It provided further evidence that the oligomerization of postfusion Ss observed in our dataset was not accidental.

During SARS-CoV-2 infection, the HR1 and HR2 domains interact with each other to bring the viral and cellular membranes close enough to form fusion pores, which makes 6-HB an important target for the development of viral entry inhibitors. We noticed that our previously reported crystal structure of SARS-CoV-2 S 6-HB (5, 7) fits well in our in situ map, suggesting that 6-HB is highly stable even on the membrane of natural viruses (*SI Appendix*, Fig. S5). We also found that, in the absence of the HR2 motif and its glycosylation, the HR1 trimer could be exposed completely outward and is accessible for entry inhibitors such as peptides and compounds. This is additional structural evidence that supports our previous design of a viral inhibitor, the highly potent pancoronavirus fusion inhibitor EK1C4, that can inhibit infection by SARS-CoV-2 and other known human coronaviruses (7). Most recently, the crystal structure and the preclinical evaluation of these HR1-targeting fusion inhibitors were reported, supporting further clinical development of these pan-CoV fusion inhibitors against SARS-CoV-2 (22). A similar strategy has been used to develop inhibitors for other viruses, for example, HIV-1 (23), LASV (Lassa virus) (24), and MERS-CoV (25).

It should also be noted that the glycosylation site N1158 is situated right at the binding site of S2P6, which is a broad neutralizing antibody that blocks membrane fusion of β-coronaviruses (26). N1158 glycosylation might play a role in shielding the binding site of S2P6 and thus allow the formation of 6-HB. Therefore, the future design of 6-HB–targeting antibodies to block viral infection should pay more attention to the steric hindrance from glycosylation sites in postfusion S of SARS-CoV-2.

As a widely used inactivation reagent to manufacture viral vaccines, BPL can not only chemically modify nucleic acids but also, to some extent, cause effects on viral proteins (27, 28). It has also been reported that BPL treatment could inhibit the membrane fusion process of the influenza virus by altering the structures and functions of viral proteins (29). However, in the present study, our BPL inactivation and sample preparation procedure maintained a reasonable population of Ss in the prefusion state. Therefore, inactivation reagents should not be the only reason to induce the conformational transition from the prefusion to postfusion state. Other factors should exist in the sample preparation procedure, which would encourage us to further optimize the inactivation strategy for better vaccine development.

In summary, our present work proposes a higher-resolution in situ structure of postfusion S of SARS-CoV-2 and discovers its oligomerization states on the membrane that possibly have important functions in the viral infection process, providing further structural information for the development of the next generation of vaccines and viral entry inhibitors.

## Materials and Methods

### Facility and Ethics Statements.

All experiments with live SARS-CoV-2 viruses were performed in the BSL-3 (P3+) facilities in the Academy of Military Medical Sciences, China. All experiments were carried out in accordance with the Regulations in the Guide for the Ministry of Science and Technology of the People’s Republic of China.

### Virus Purification and Cryo-EM Tomography Sample Preparation.

Vero cells (American Type Culture Collection, CCL-81) were maintained in Dulbecco’s modified essential medium supplemented with 10% fetal bovine serum (Biowest) at 37 °C with 5% CO_2_. The strain BetaCoV/Wuhan/AMMS01/2020 was originally isolated from a COVID-19 patient returning from Wuhan, China. The virus was amplified and titrated by a standard plaque-forming assay on Vero cells, as previously reported (30, 31). SARS-CoV-2 was cultured in large-scale Vero cell factories at a multiplicity of infection of 0.5 at 37 °C with 5% CO_2_. To inactivate virus production, BPL was thoroughly mixed with the supernatant of the infected cells at a ratio of 1:4,000 vol/vol for 48 h at 2 °C to 8 °C. Following clarification of the cell debris and ultrafiltration, the inactivated viruses were purified by ion exchange chromatography and size exclusion chromatography, as previously reported (32, 33). Purified viruses were mixed at a ratio of 5:1 (virus:gold) with 10 nm of protein A-coated gold fiducials (Electron Microscopy Sciences). Then, 3 μL of the mixture was applied onto a discharged 300 mesh copper grid with a C-flat R 2/1 holey carbon support film. Grids were blotted for 3 s in 100% relative humidity for plunge freezing in liquid ethane using Vitrobot (Thermo Fisher Scientific).

### Cryo-ET Data Acquisition.

Cryogrids were loaded into an FEI Titan Krios G2 transmission electron microscope (Thermo Fisher Scientific) operated at 300 kV, and images were recorded on a Gatan K2 Summit DDD camera (Gatan Company) in superresolution mode equipped with a Gatan Quantum energy filter with a slit width of 20 eV in zero-loss mode. Nominal magnification was set to 105,000×, resulting in a calibrated physical pixel size of 1.36 Å at the specimen level. Tilt series between −60° and +60° were acquired using a dose-symmetric scheme with a 3° angular increment using SerialEM software with an in-house script (34). A total dose of 123 e^−^/Å^2^ per tilt series was distributed evenly among 41 tilts. The defocus range was set between −1.5 μm and −3 μm, and 10 frames were saved for each tilt angle.

### Image Processing and Subtomogram Averaging.

The output superresolution movies were first subjected to motion correction with a binning level of two using Warp (35), resulting in a pixel size of 1.36 Å, and masking of fiducial markers was also performed using boxnet tools inside Warp. All 373 tilt series stacks were generated using automatic procedures in Warp. To gain the best tilt series alignment quality, only tilts within −45° to +45° were kept for further alignment. Alignments of tilt series were performed using automatic tilt series alignment functions in the Dynamo (a software environment for subtomogram averaging of cryo-EM data) and IMOD (a computer software package for analyzing and viewing three-dimensional biological image data) packages (36–38). The tilt series with completely failed alignments were discarded by visual inspection using IMOD (38). Then, the alignment files of 352 successfully aligned tilt series were transferred back to Warp to perform per-tilt contrast transfer function (CTF) estimation. Tomograms were reconstructed in Warp at a binning level of eight and deconvolved for better visualization.

For particle picking of prefusion Ss, we used a previously reported cryo-EM map of prefusion S (EMD-21452) (8) low-pass filtered to 40 Å as a reference for template matching by the Dynamo package (37) in eight binned deconvolved tomograms. Postfusion Ss were manually picked using Dynamo packages (37), whose initial Euler angles (two out of three) were determined based on the vector between two manually set points, one in the middle of S and another on the membrane. Then, the coordinates and orientations of 7,656 prefusion and 7,869 postfusion particles were employed for the extraction of subvolumes in Warp with 48 × 48 × 48 voxels at a voxel spacing of 10.88 Å. The corresponding 3D CTF models were also generated considering accumulated radiation damage. To determine the exact ratio of prefusion and postfusion states on a nonbiased basis, an average of all extracted prefusion and postfusion Ss was generated to be used as a single reference for 3D classification in RELION (a computer program for cryo-EM data processing) versions 3.0 and 3.1 (39, 40). The classification converged into four different groups: one exhibited a strong density of double-layer membranes (10%), two exhibited the typical morphology of postfusion S (48%), and one exhibited the typical morphology of prefusion S (42%). Using the above classified prefusion coordinates and orientations of the Ss, we performed particle reextraction with a binning level of two using Warp. A subsequent autorefinement job against 6,456 particles using RELION yielded a 12.9-Å map of prefusion S (*SI Appendix*, Fig. S2).

To achieve a high-resolution map of postfusion S without model bias, no prior structures or maps from other studies were used throughout the data processing steps. First, the subtomograms were directly averaged without alignment and symmetry applied to generate a data-driven low-resolution template with only manually set Euler angles applied using relion_reconstruct. This process yields a good reference for subsequent alignment (*SI Appendix*, Fig. S2). To validate the applied symmetry and find a good reference for further alignment, four different sessions with or without restrictions on the search range for rot. and tilt angles and with or without the threefold symmetry applied were performed. These sessions converged into an average with observed threefold symmetry and with the double layer of viral membrane along with TM domain visible. However, only one session with a restricted search for rot. and tilt angles and with threefold symmetry applied resulted in higher resolution at the same binning level. Using this refinement setup, all particles were further aligned with a mask solely covering the connector domain and 6-HB domain, which resulted in an average map with a resolution of 21.76 Å at a binning level of eight. Then, particle reextraction was performed using Warp with a binning level of four. Another round of local refinement was performed in RELION, yielding an average map with a resolution of 12.7 Å. Then, particle reextraction was performed again using Warp with a binning level of two. In the subsequent local refinement, particles with a shift greater than the mean ±2× SD were discarded, which resulted in 5,463 particles and a final map with a resolution of 10.9 Å. Postprocessing in RELION with visually estimated B factor was applied for map sharpening (*SI Appendix*, Figs. S2 and S6).

### Distance Calculation of the Nearest Pair Distances.

For postfusion Ss, the centers of 2,771 virions that possessed postfusion Ss on their membranes were manually picked from the deconvolved tomograms. All the postfusion Ss were assigned to the corresponding virions. For virions that possessed at least two postfusion Ss, we calculated the nearest pair distance for each S protein as follows. We selected one S protein at a time, calculated the distances between its center and the center of every other S protein on the same virion, and then found the minimum distance, which was defined as the nearest pair distance for that S protein. This value was calculated for all the S proteins, and the results were plotted into a histogram (Fig. 1*F*). The nearest pair distances for prefusion Ss were calculated in the same way (*SI Appendix*, Fig. S3*B*).

For the simulated dataset, randomly distributed Ss were placed on imaginary sphere-shaped virus with a diameter of 50 nm. The number of viruses and the number of Ss on each virus were all kept the same as the experimental data for postfusion Ss. Then the nearest pair distances were calculated in the same way (Fig. 1*F*).

### Calculation of Distance between Postfusion Ss and AWI.

We manually marked the upper and lower surfaces of AWI using the slicing tool in IMOD (38) by viewing tomograms from the *y*–*z* plane. Based on the *z* axis coordinates of particles and the AWI coordinates of their tomogram, the relative *z* axis positions can be calculated (*SI Appendix*, Fig. S3*C*). The average thickness of all tomograms used in this study had an average thickness of about 120 nm.

### Model Fitting and Data Analysis.

The previously reported postfusion structures of the purified SARS-CoV-2 S protein (PDB entries: 6XRA and 6LXT) (5, 15) were fitted to the map. The extended parts were built manually using COOT (a molecular graphics application) (41). The final model was refined according to the map using PHENIX.Refine (42). Visualization and model analysis were performed with UCSF (University of California, San Francisco) Chimera (43) and UCSF ChimeraX (44). Cross-correlation (CC) values of the model to map were calculated by PHENIX (45) to 0.73 and 0.77 for CC(mask) and CC(box), respectively.

## Supplementary Material

Supplementary File

Supplementary File

Supplementary File

## Data Availability

The atomic model of this study has been deposited in the RCSB (Research Collaboratory for Structural Bioinformatics) PDB under accession code 7E9T. The electron density map from this study has been deposited in the Electron Microscopy Data Bank under accession code 31037. All raw tilt series used in this study has been deposited in EMPIAR (the Electron Microscopy Public Image Archive) China (http://www.emdb-China.org.cn) under accession code EMPIARC-200001.
