# Supplementary material for: Nanometer-resolution in situ structure of the SARS-CoV-2 postfusion spike protein
Source: Proc Natl Acad Sci U S A. 2021 Nov 15;118(48):e2112703118. doi: 10.1073/pnas.2112703118 (PMC8640741; doi:10.1073/pnas.2112703118)
Supplement: Supplementary File [file pnas.2112703118.sapp.pdf]

PNAS
www.pnas.org

Supplementary Information for
Nanometer resolution *in situ* structure of the SARS-CoV-2 postfusion spike protein

Linhua Tai[1,2,*], Guoliang Zhu[1,2,*], Minnan Yang[1*], Lei Cao[1], Xiaorui Xing[1],Guoliang Yin[1,2], Chun Chan[4], Chengfeng Qin[5], Zihe Rao[1], Xiangxi Wang[1,6,#], Fei Sun[1,2,3,6,#], Yun Zhu[1,#]

#Correspondence: Yun Zhu (zhuyun@ibp.ac.cn), Fei Sun (feisun@ibp.ac.cn), Xiangxi Wang(xiangxi@ibp.ac.cn)

This PDF file includes:

Figures S1 to S6
Table S1
Legends for Movies S1 to S2

Other supplementary materials for this manuscript include the following:

Movies S1 to S2

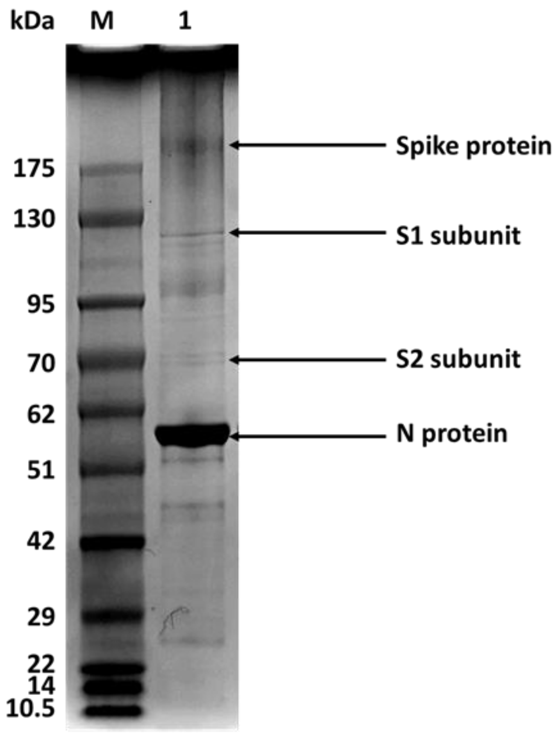

Fig. S1. SDS-PAGE of purified the SARS-CoV-2 virions used in this study. Lane M: protein marker, indicating different molecular weights. Lane 1: purified virions, indicating the different protein components.

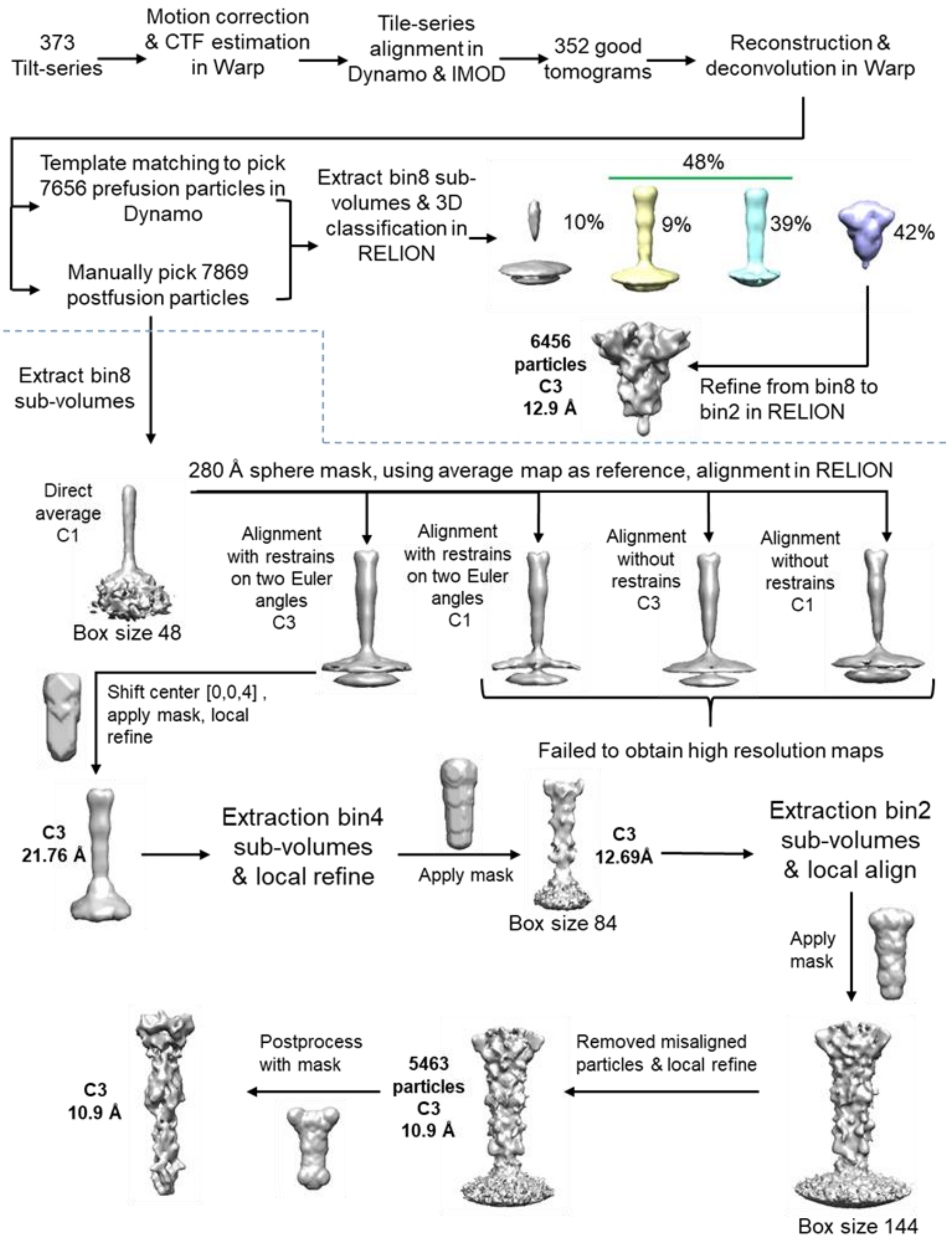

Fig. S2. A flow chart showing the processing of the cryo-ET dataset and subtomogram analysis of SARS-CoV-2 S in both the pre- and postfusion states. For details, see **Materials and Methods.**

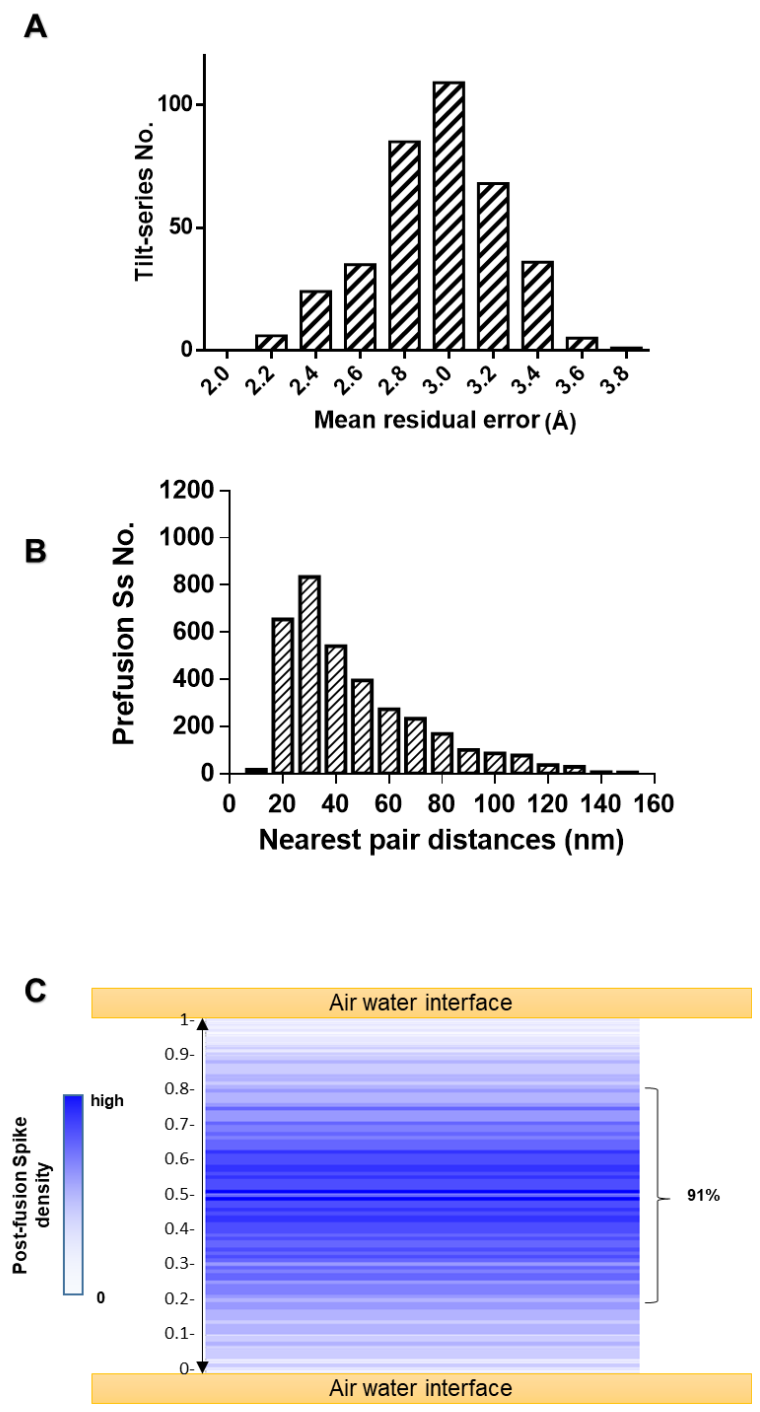

Fig. S3. The statistics of tilt series alignments and Ss' distributions. (A) The histogram of alignment errors for the 352 cryo-ET tilt series. **(B)** The histogram of the nearest pair distances of prefusion Ss. (**C**) The distribution of relative positions of postfusion Ss in the Z-axis of reconstructed tomograms.

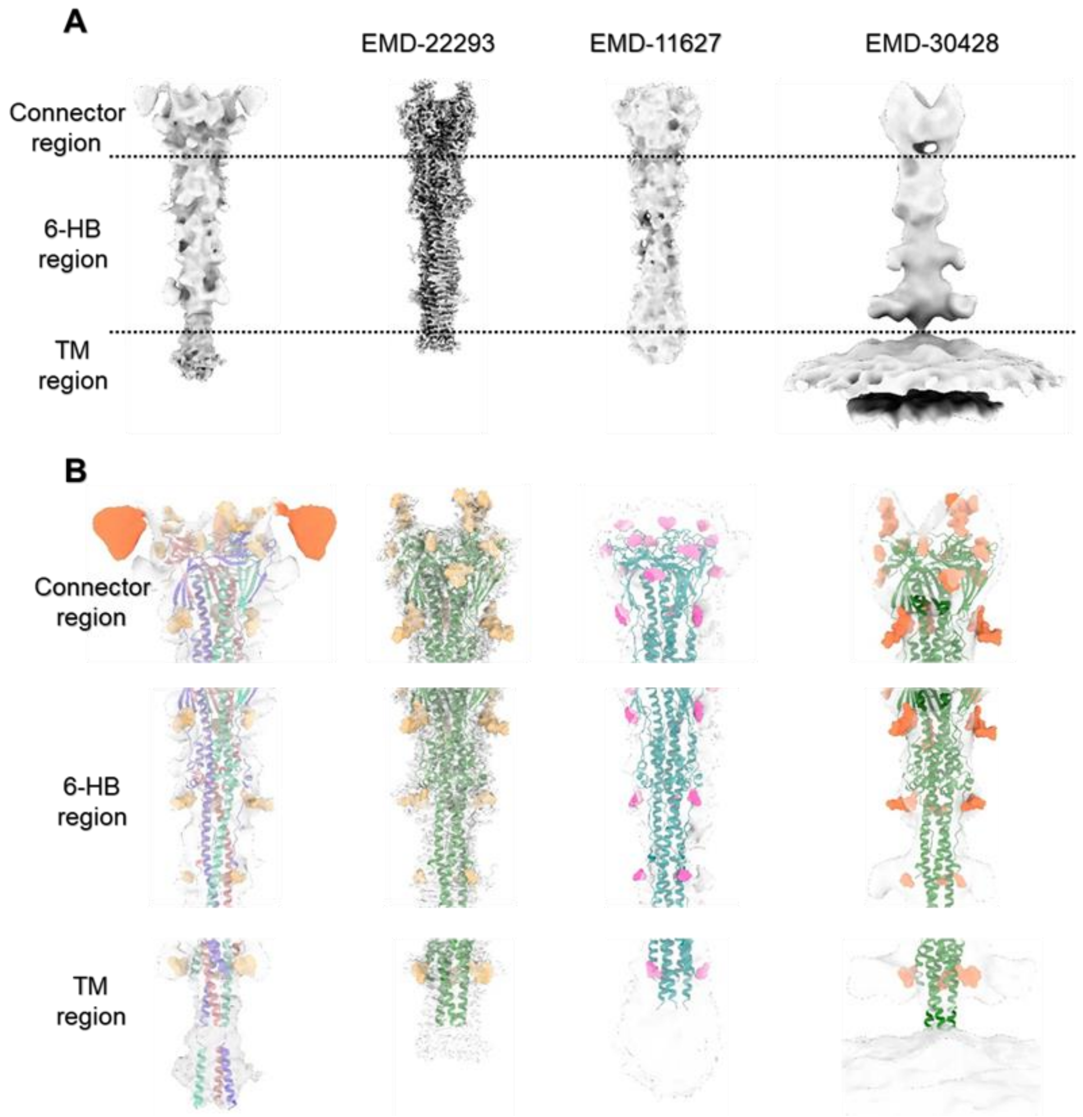

Fig. S4. Comparing the map quality and model completion of this study with other published results. (A) Our map of postfusion S was aligned side by side with three reported maps (EMD-22293, EMD-11627, EMD-30428). The different regions are marked with dotted lines and indicated. **(B)** Our model of postfusion S was compared with three reported models (PDB entry 6XRA for EMD-22293, PDB entry 6M3W for EMD-11627, and PDB entry 6XRA for EMD-30428). In our model, the coloring scheme is the same as that in Fig. 3C, and unassigned densities are colored coral. In the other models, the proteins are colored forest green and dark cyan for PDB 6XRA and 6M3W, respectively, and glycosylation sites are colored chocolate, hot pink and coral in EMD-22293, EMD-11627 and EMD-30428, respectively. All maps are displayed using the recommended contour level available on EMDB entry, and fitting of their relative models was performed using UCSF Chimera. All maps and models are displayed and aligned on the same scale.

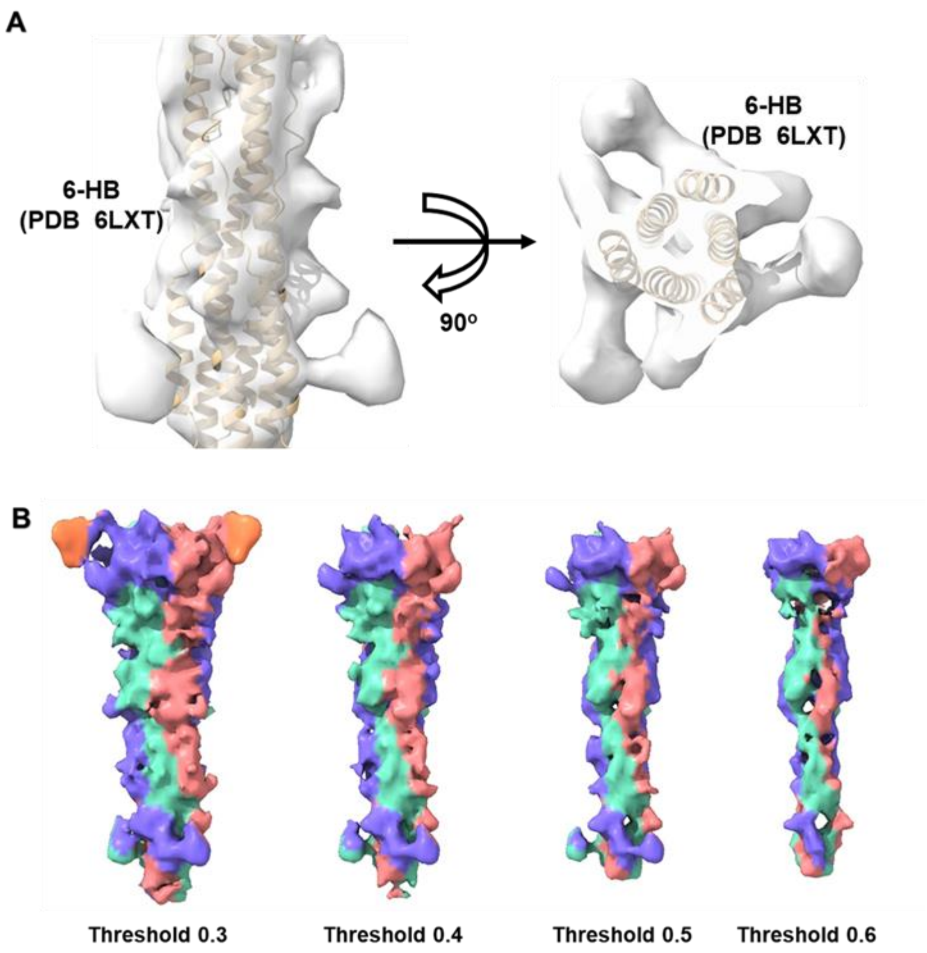

Fig. S5. Our *in situ* map of postfusion S fitted by the crystal structure of 6-HB and shown at different threshold levels. (A) The crystal structure of SARS-CoV-2 S 6-HB (PDB entry 6LXT) is shown as a cartoon in wheat, and the fitting map is shown in both the side view (left) and top view (right). (**B**) The maps at different threshold levels are colored the same as in Fig. 2A, and unassigned densities are colored coral.

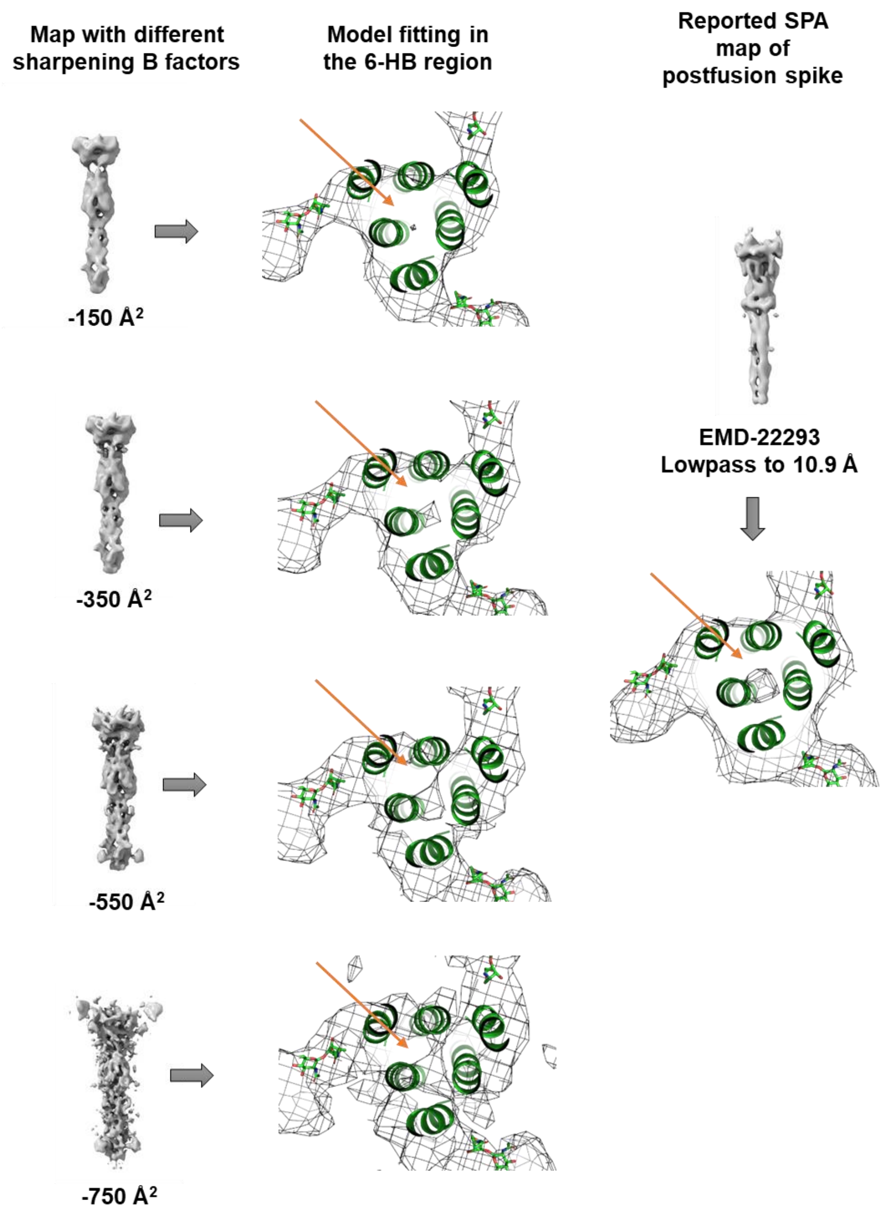

Fig. S6. Estimations of various sharpening B factors of postfusion Ss.

Table S1. Statistics of cryo-ET data collection and image processing.

**Data Collection**	
Microscope	Titan Krios G2
Voltage (kV)	300
Detector	Gatan K2
Energy filter	Gatan GIF Quantum, 20 eV
Mode	Super-resolution
Pixel size (Å)	0.68
Tilt step & range	-60° ~ +60°, 3° step, start at 0°
Tilt scheme	Dose-symmetric
Number of tilt-series	373
Exposure per tilt (e^-/Å^2)	3
Number of frames per tilt	10
Defocus range (µm)	-1.5 ~ -3
Software	SerialEM
**Reconstruction**	
Software	Dynamo v1.1.509<br>Relion v3.0 & 3.1<br>Warp 1.0.7 & 1.0.9
Data set	Postfusion S protein
Final number of particles	5463
Symmetry	C3
Final resolution (Å)	10.9
Map pixel size (Å)	2.72
Map sharpening B-factor (Å^2)	-350

Movie S1 (separate file). Overview of the cryo-STA map and model fitting of SARS-CoV-2 postfusion S.

Movie S2 (separate file). Possible mechanism of the SARS-CoV-2 S transition from the prefusion state to the postfusion state during the membrane fusion process.
